# Supplementary material for: Enhancing trauma cardiopulmonary resuscitation simulation training with the use of virtual reality (Trauma SimVR): Protocol for a randomized controlled trial
Source: PLoS One. 2025 Jan 24;20(1):e0316828. doi: 10.1371/journal.pone.0316828 (PMC11761589; doi:10.1371/journal.pone.0316828)
Supplement: S2 File — (PDF) [file pone.0316828.s002.pdf]

Study protocol

Enhancing Trauma Resuscitation Simulation Training with the Use of Virtual Reality (Trauma SimVR Study)

Version 1.4 / March 22nd, 2024

Josef Michael Lintschinger[1,2], Philipp Metelka[1,2], Lorenz Kapral[1], Florian Kahlfuss[2], Lena Reischmann-Senoner[1], Caroline Holaubek[2,3], Georg Kaiser[4], Alexandra Kaider[5], Eva Schaden[1,2], Christina Hafner[1,2,3]

[1] Ludwig Boltzmann Institute Digital Health and Patient Safety; Waehringer Straße 104/10, A-1180 Vienna

[2] Department of Anesthesia, General Intensive Care Medicine and Pain Medicine, Medical University of Vienna; Waehringer Guertel 18-20, A-1090 Vienna

[3] Medical Simulation Center, Medical University of Vienna; Brünner Straße 68, A-1210 Vienna

[4] Department of Orthopedics and Trauma Surgery, Medical University of Vienna, Vienna, Austria

[5] Center for Medical Data Science, Medical University of Vienna, Vienna, Austria

SYNOPSIS

**Title**	Enhancing Trauma Resuscitation Simulation Training with the Use of Virtual Reality (The Trauma SimVR Study)
**Protocol date**	March 22nd, 2024
**Protocol version**	1.4
**Ethics committee number**	1388/2023
**ClinicalTrials.gov identifier (NCT number):**	NCT06445764
**Hypothesis**	We hypothesize that the use of VR simulation in the context of trauma CPR training will lead to improved performance in the management of trauma patients going into cardiac arrest, with greater confidence in clinical decision-making, resulting in shorter times to order/perform critical actions.
**Primary aim**	The primary aim of this study is to evaluate the effectiveness of using VR technology, as opposed to e-learning preparation, to equip learners with the skills and knowledge necessary for in-person simulation training and to improve their performance in TCA management skills.
**Secondary aim**	The secondary aims are to assess gender differences in performance, the frequency of protocol deviations, the cognitive load, and the gaze behavior between groups as well as participants' acceptance and impressions of VR in medical education.
**Design**	Single-center, prospective, single-blind, randomized, controlled trial
**Intervention**	VR simulation training for managing trauma patients going into cardiac arrest
**Control**	Training/preparation for the management of trauma patients going into cardiac arrest using an e-learning course
**Sample size**	67
**Inclusion criteria**	Junior physician (common-trunk) or first-year resident in the field of anesthesiology, intensive care, emergency medicine or traumatology at the Medical University of Vienna; only people who do not need eyeglasses for using VR; ≥ 18 years
**Exclusion criteria**	Pre-disposition for cybersickness (motion sickness, pregnancy, pre-existing cybersickness)
**Drop-out criteria**	Use of any other theoretical or hands-on trauma and/or CPR training opportunities outside those given in the respective randomized group during study participation; incomplete preparation with the assigned study method

CPR = cardiopulmonary resuscitation; VR = virtual reality

LIST OF ABBREVIATIONS

Abbreviation	Definition
ACLS	Advanced cardiac life support
AE	Adverse event
AHA	American Heart Association
ALS	Advanced life support
AOI	Area of interest
ATLS	Advanced trauma life support
BLS	Basic life support
CASTesting	Cardiac arrest simulation testing
CPR	Cardiopulmonary resuscitation
CRM	Crew resource management
ERC	European Resuscitation Council
eFAST	Extended Focused Assessment with Sonography for Trauma
$etCO_2$	End-tidal carbon dioxide
HCP	Healthcare providers
ID	Identfication
IHCA	In-hospital cardiac arrest
ILCOR	International Liaison Committee of Resuscitation
MedUni Vienna	Medical University of Vienna
NASA-TLX	National Aeronautics and Space Administration Task Load Index
PEA	Pulseless electric activity
POCUS	Point-of-care ultrasound
ROSC	Return of spontaneous circulation
spO_2	Oxygen saturation
SUS	System usability scale
TCA	Traumatic cardiac arrest
VR	Virtual reality

TABLE OF CONTENTS

SYNOPSIS 2

LIST OF ABBREVIATIONS 3

1 BACKGROUND 6

2 RESEARCH HYPOTHESIS AND OBJECTIVES 7
2.1 Hypothesis 7
2.2 Primary Objective 7
2.3 Secondary Objectives 7

3 Methodology 9
3.1 Study population 9
3.1.1 Inclusion Criteria 9
3.1.2 Exclusion criteria 9
3.1.3 Drop out criteria 9
3.2 Study design 10
3.2.1 Randomization 12
3.2.2 Control and Intervention 12
3.2.3 Simulation sessions 13
3.2.4 Performance assessment 17
3.3 Sample size 19
3.4 Data collection 19
3.4.1 Variables 19
3.4.2 Data protection 21
3.4.3 Data analysis 22
3.5 Statistical methods 22
3.6 Materials 23
3.6.1 VR Headset 23
3.6.2 Eye-tracking glasses 23
3.6.3 E-learning platform 24
3.6.4 Video editing 24

4 PROTECTION OF HUMAN SUBJECTS 25

4.1 Informed consent 25
4.2 Patient withdrawal 25
4.3 Ethical considerations and risk-benefit-evaluation 25

REFERENCES 26

APPENDIX 30

1 BACKGROUND

Traumatic cardiac arrest (TCA) is an incredibly devastating condition with a low survival rate (1) that requires urgent recognition, rapid intervention, and specialized resuscitation skills in both in-hospital and out-of-hospital settings. In Austria, as in many other countries, the transfer of prehospital trauma patients to specialized emergency departments, so-called shock rooms, staffed by anesthesiologists and traumatologists is critical because they require highly specialized interventions such as bleeding control, airway management, patient blood management, etc. and patients are often at risk of cardiac arrest upon arrival. However, in-hospital cardiac arrest (IHCA) care has been shown to be often substandard and not fully consistent with international guidelines (2–5). Therefore, the International Liaison Committee of Resuscitation (ILCOR) and the American Heart Association (AHA) recommend focused cardiopulmonary resuscitation (CPR) training for IHCA as a primary area of interest to improve survival from IHCA (6,7).

Simulation is the gold standard for training various medical scenarios in a safe and forgiving environment (8–10), with an additional focus on patient safety (9,11). This is particularly important in the context of rare but life-threatening conditions (8,11,12) such as TCA. In contrast, it is important to note that the highly specialized skills required to manage TCA may not be commonly taught in standard adult advanced life support (ALS) courses and simulations. In addition, the procedures required for TCA management deviate to some extent from standard ALS care, as suggested by the European Resuscitation Council (ERC) and are rarely used in daily practice. As a result, healthcare providers (HCPs) need to undergo rigorous training (13–15) to ensure that they have the necessary skills and expertise to effectively manage TCA patients when needed. Although simulation-based training is an effective way to improve trauma resuscitation skills, the complexity of TCA scenarios and the need for specialized equipment and trained personnel often limit simulation opportunities and make them very expensive and rare. This could lead to poor training outcomes and reduced patient survival rates.

To address this issue, innovative, less expensive, and more accessible training methods need to be developed. Virtual reality (VR) simulation is an emerging approach that enables realistic and immersive training scenarios without the need for expensive equipment or physical space (11,16–18). In-situ simulation, on the other hand, allows for training in the actual clinical environment, allowing for the assessment of team dynamics and human factors that may affect resuscitation outcomes. The combination of pre-course VR preparation (15,19) and in-situ simulation approaches (15,19–21) could ultimately improve the TCA management skills of HCPs and also have a positive impact on the outcomes of critically injured patients. Blended learning approaches appear to provide the best educational value for CPR training (15,20,22–24).

Therefore, the primary aim of this study is to evaluate the effectiveness of using VR technology, as opposed to e-learning (19,25–27) preparation, to equip learners with the skills and knowledge necessary for in-person simulation training and to improve their performance in TCA management skills. The secondary aims are to assess gender differences in performance (28,29), the frequency of protocol deviations, the cognitive load, and the gaze behavior between groups as well as participants' acceptance and impressions of VR in medical education.

2 RESEARCH HYPOTHESIS AND OBJECTIVES

2.1 Hypothesis

We hypothesize that the use of VR simulation in the context of trauma CPR training will lead to improved performance in the management of trauma patients going into cardiac arrest, with greater confidence in clinical decision-making, resulting in shorter times to order/perform critical actions.

2.2 Primary Objective

The primary objective of this study is to evaluate whether a VR-based approach is superior to an e-learning-based approach in the context of trauma CPR training and preparation prior to testing trainee performance in an in-person simulation session. The primary outcome measure is the time from the start of chest compressions to critical action #3 *(mini-thoracotomy;* Table 1) in the simulation (assessment) session.

2.3 Secondary Objectives

1) Comparing the number of participants who did not recognize and treat the underlying condition (one or more missed critical actions) of the TCA between randomized groups.
2) Comparing the number of participants who prematurely declared a patient dead (before the 15-minute time limit of the scenario).
3) Comparing the time to critical actions, excluding the primary outcome measure, between randomized groups.
4) Comparing the proportions of the respective protocol deviations between randomized groups.
5) Evaluate gender differences in the primary outcome.
6) Comparison of cognitive load between the randomized groups while performing the simulated assessment scenarios as measured by the National Aeronautics and Space Administration Task Load Index (NASA TLX).
7) Eye-tracking
    a) Comparing the gaze behavior (dwell-time in AOIs, fixation count, and average fixation duration) between randomized groups.
    b) Comparing the relative time when no AOI is illustrated between randomized groups.
8) Using a 5-point Likert scale:
    a) Participants' subjective impressions of their learning progress when using VR/e-learning.

b) Participants' subjective impressions of their level of frustration when using VR/e-learning.

c) Participants' subjective impression of their level of enjoyment when using VR/e-learning.

d) Participants' subjective confidence in recognizing and providing initial care to polytraumatized patients in cardiac arrest in the simulation sessions.

e) Participants' subjective overall performance in the simulation sessions.

f) Participants' overall performance in the simulation sessions from the expert's point of view.

9) To assess the correlation between how often participants have played VR and non-VR video games in the past 12 months and between the ages of 6 and 18 and the primary outcome?

10) The incidence rate of VR-related adverse events (AEs).

11) System usability scale (SUS) for the use of the VR simulations.

3 Methodology

3.1 Study population

The focus of this study is on junior physicians in the so-called common-trunk phase, who have already completed their medical studies and are working as physicians but are not yet enrolled in a definitive residency program, as well as first-year residents in anesthesiology/intensive care, emergency medicine, and traumatology at the Medical University of Vienna (MedUni Vienna).

Participants will be recruited through bulletin board postings (Additional file #5), email lists, and social media channels of MedUni Vienna.

3.1.1 Inclusion Criteria

To be eligible for this study, participants must meet all of the following inclusion criteria:

- Junior physician (common-trunk) or first-year resident in the field of anesthesiology, intensive care, emergency medicine, or traumatology at MedUni Vienna
- Only people who do not need eyeglasses for using VR
- ≥ 18 years

3.1.2 Exclusion criteria

Participants meeting the following criteria will not be included in the study:

- Pre-disposition for cybersickness (motion sickness, pregnancy, pre-existing cybersickness)

3.1.3 Drop out criteria

Participants will be withdrawn from the study if any of the following criteria are met:

- Use of any other theoretical or hands-on trauma and/or CPR training opportunities outside those given in the respective randomized group during study participation
- Incomplete preparation with the assigned study method

3.2 Study design

This study is a single-center, prospective, single-blind, randomized, controlled trial. It aims to assess the efficacy of VR as a more effective preparation method for in-person simulation trainings, compared to e-learning. The focus is on improving physicians' trauma CPR management skills.

All recruited participants will watch a 60-minute video to learn about the requirements, steps, and other important information about this study. Furthermore, this introductory video will give an overview of management strategies for trauma patients going into cardiac arrest in an in-hospital setting and provides links to the guidelines (30–32) on which this study is based. It also indicates that knowledge of the content of the guidelines is a prerequisite for Phases 2 through 3. This video can be viewed as often as desired throughout the study. Participants will also be required to complete the electronic Questionnaire 1 (Additional file #2) prior to randomization, which assesses participants' baseline characteristics (including the German versions of the "Openness to Experience" subscale of the 100-item HEXACO-PI-R test (33,34) and the Technology Readiness Scale (35)).

Each participant will then complete either the e-learning course (control group) or the VR training simulations (intervention group) over a two-week period, both focusing on the same content. Both groups will spend approximately the same amount of time learning the same content, which will prepare the participants for the assessment in the simulation session. If participants do not complete the entire e-learning course or all four VR simulations, they will be dropped from the study.

After Phase 2, all participants will attend the simulation session and will be evaluated in the role of medical team leader of a standardized TCA scenario (Phase 3).

In addition, each participant will be required to complete Questionnaire 2 (Additional file #3) immediately following the simulation session in Phase 3. Only the intervention group will be required to complete the VR system usability portion of this questionnaire. The system usability scale (SUS) (36,37) will be translated and used in German.

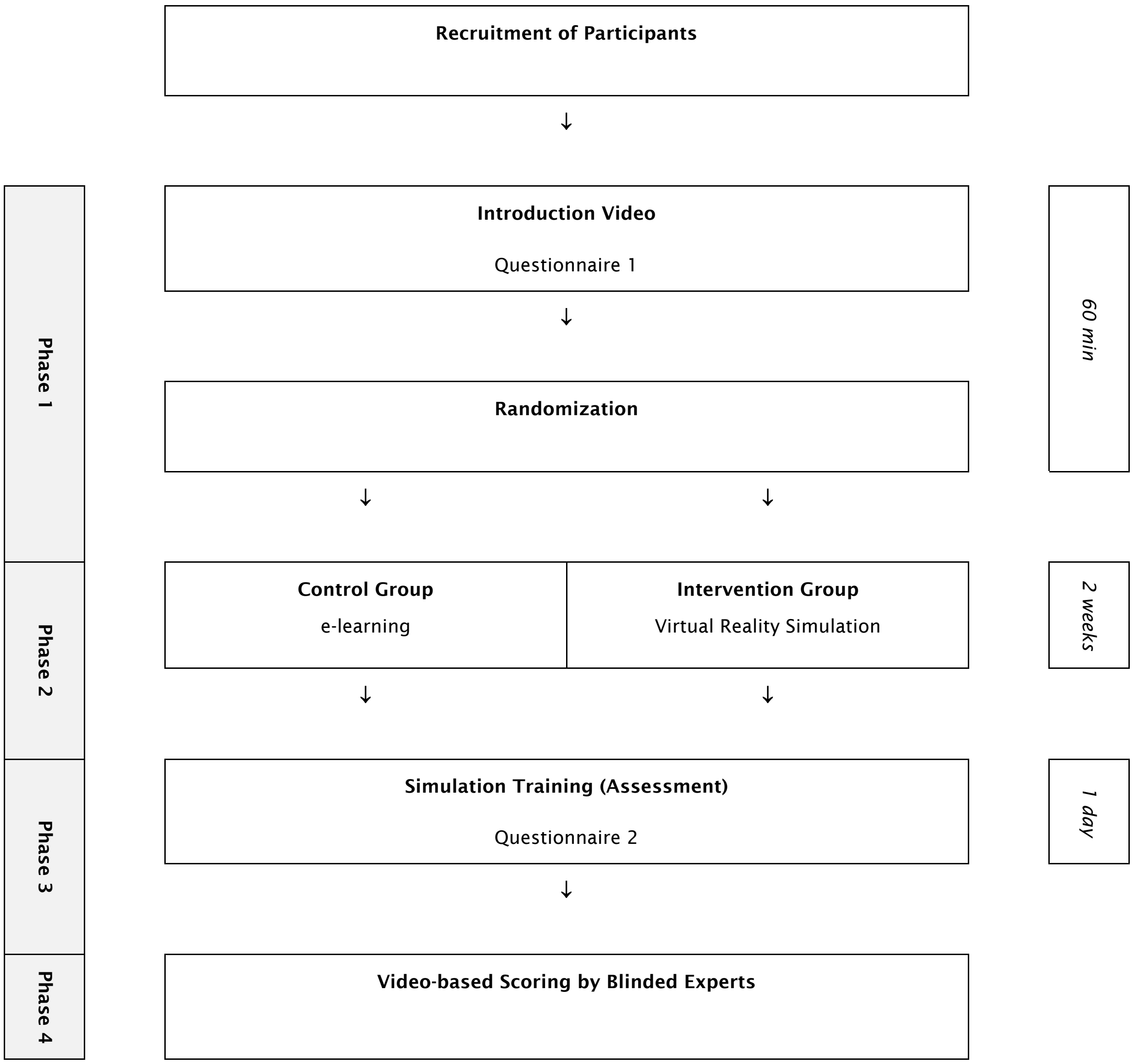

Figure 1. The course of events during the study.

3.2.1 Randomization

Recruited participants will be randomly assigned to the control or intervention group on an equal basis by gender. Participants will not know their allocation to either group until they have watched the entire introductory video and completed Questionnaire 1. Randomization will be performed using the web-based randomization software Randomizer from MedUni Vienna (38).

3.2.2 Control and Intervention

The control group will complete an e-learning course over a two-week period in preparation for the subsequent simulation session, which will teach the same content as the VR simulation software in the intervention group.

Both e-learning and VR have the limitation that manual skills cannot be practiced. The content will therefore focus on procedural processes and guideline adherence in the context of trauma patient care and traumatic cardiac arrest in shock rooms. In addition to basic life support (BLS) and ALS, there will be additional specific learning content (15) such as structured patient assessment (xABCDE approach) and special care strategies for this unique patient population.

The e-learning course will also focus on basic principles of crew resource management (CRM) and related communication skills (15,39), such as closed-loop communication. It has also been shown that cognitive aids such as checklists, flowcharts, or mnemonics can be very helpful to HCPs in such settings, and their use is recommended (20). Accordingly, their use will also be an essential part of this e-learning course.

Once enrolled, all participants randomized to this group will receive access information for the course, which, like the VR training, will be conducted in German and must be completed within 2 weeks.

Participants randomized to the intervention group will learn and practice the exact same content using a VR training environment co-developed by the research team of this study. The developed software with its four TCA scenarios must also be practiced over a period of 2 weeks, which corresponds to the same time and learning effort as the e-learning course.

In order to make the VR training sessions as efficient as possible, it is necessary to provide the participants with an additional orientation module that must be played before the VR training sessions begin. This additional module will take about 20 minutes to play once to familiarize the participants with the technical features of the VR headset and the controllers and to keep their focus on the medical content instead of the technical features. The developed software will be a single-user application with automated agents as a resuscitation team for interaction.

All four virtual intervention group simulations or the entire e-learning course must be completed before entering Phase 3, or the participant will be dropped from the study.

VR-related adverse events:

AEs may occur during the use of VR, especially cybersickness (41). The following symptoms' incidence will be evaluated accordingly:

- Nausea and/or vomiting
- Dizziness
- Headaches
- Eye strain and fatigue (discomfort, blurred vision, reduced visual acuity)
- Physical effects (tripping, falling, and bumping into objects in the real world)

3.2.3 Simulation sessions

The simulation sessions (assessments) in Phase 3 will take place in the simulation rooms of MedUni Vienna. Before beginning the manikin-based simulation scenario, participants will be given time to orient themselves and become familiar with the room and the setting itself, the various functions of the manikin, the equipment, and the medications provided. The positioning of the manikin and the equipment will intentionally be laid out to look as similar as possible to the illustrations provided in the e-learning course and to the VR environment.

Participants act as medical team leaders to complete a standardized trauma scenario, which is not known in advance, in a simulated shock room. However, it is defined that the participants know beforehand that basically all the content of the preparatory courses/simulations in Phase 2 could be of importance in the simulated scenario. Which of these learned and trained skills will be used when, if, and how is entirely up to the participants.

The corresponding assessment scenario, which is identical for both groups, refers to a trauma patient with a developing tension pneumothorax who will always require resuscitation. Such an approach is also consistent with the ALS assessment standards of the so-called CASTesting (cardiac arrest simulation testing) in the ERC provider courses. In this study, however, an additional focus is placed on the care of critically injured trauma patients entering a resuscitation situation in a shock room setting. Because of this additional focus, these scenarios will last for 15 instead of 10 minutes, which is ERC's standard for CASTesting (42).

In order to objectively evaluate the participants' performance, the scenario will be recorded by pre-installed cameras in the simulation room. In addition, all actions will be recorded from the participant's perspective using eye-tracking glasses.

The simulation room provides nearly identical conditions and materials as the real shock room. The high-fidelity manikin used will be controlled in a predefined and standardized manner from the control room by experts on the study team.

The physician being assessed always enters the scenario alone. In this setting, seven people form a team and only the team leader is evaluated. The other six confederates, members of the study team, will

represent one resident in anesthesiology and intensive care, one resident and one specialist in traumatology, two nurses, and a radiology assistant. They will act naturally and only as an emergency/resuscitation team. These team members are not allowed to assist the team leader with medical decisions and are only allowed to perform actions on the team leader's orders.

The scenario used begins in a shock room setting with the patient already transferred from the ambulance rescue stretcher to the shock room table and will already be fully monitored. The patient will already have a small lumen (20G) peripheral venous catheter and will be ventilated with an endotracheal tube. Prior to the start of the scenario, all further information regarding the patient's rescue by the emergency medical service will be provided by the emergency physician to the team leader (participant) on a sheet of paper using the SBAR approach. At this point, the patient is wearing only shorts and there is no external bleeding apparent. Immediately after the start of the scenario, which is defined as the time when the instructor gives a "start" signal, exactly 20 seconds after he/she hands over the aforementioned sheet of paper, the patient's hemodynamic deterioration will be fulminant, leading to cardiac arrest after a predefined time.

For performance assessment, critical actions (Table 1) and protocol deviations (Table 2) were defined according to the ERC guidelines on adult ALS (30) and cardiac arrest in special circumstances (31) and the AWMF guideline on polytrauma and management of critically injured patients (32).

Table 1. Critical actions

	**Critical action**	**Time measurement**	**Notes**
#1	CPR start	From the onset of cardiac arrest to the start of chest compressions	The defined point for time measurement is when a team member's hands touch the chest to begin chest compressions.
#2	Recognition of tension pneumothorax	From the scenario start to recognition of the tension pneumothorax	The defined point of time measurement is when the team leader fully pronounces one of the key words, or the earliest point of time when the evaluating expert can conclude the diagnosis from other team leader's phrases. These phrases will be documented.<br><br>**Key words**: Pneu; Pneumothorax; Spannungspneumothorax; Entlasten; Bülau; Thoraxdrainage; Minithorakotomie
#3	Mini-Thoracotomy	From the start of CPR (defined as in critical action 1) to performing the mini-thoracotomy	The point at which the scalpel touches the chest to perform the mini-thoracotomy is the defined point for time measurement.

CPR = cardiopulmonary resuscitation

Critical actions are defined as actions that, if not performed correctly and in a timely manner, could result in serious harm to the patient in this scenario. As these critical actions build on each other, the primary outcome is defined as critical action #3. The simulation (assessment) session will also not allow to perform a mini-thoracotomy before starting CPR.

The measured times to critical actions #1 and #2 will also be assessed as a secondary outcome.

If the participants do not recognize the underlying cause of the TCA, tension pneumothorax, and therefore do not direct CPR to prioritize and perform a mini-thoracotomy, a simulated specialist in anesthesiology and intensive care will enter the scenario and take over team leadership at exactly 15 minutes. In this case, the scenario will continue for an additional three minutes to achieve ROSC and still provide a positive learning outcome for the participants but will be considered a censored observation for further statistical evaluation.

The purpose of this approach is to ensure that the specific sequence of a TCA, with the increased/earlier focus on ruling out reversible causes and even interrupting chest compressions to treat them, if necessary, is the focus of CPR in these special circumstances.

Table 2. Protocol deviations

**Primary assessment**		
c	Actively searching for signs of massive internal or external bleeding	Inspection and palpation
A/B	Check airway/tube placement	Position check (etCO2, auscultation)
	Check thorax	Inspection and auscultation (if not already done)
	Check respirator settings	Checking the respirator and its settings
	Check jugular veins	Inspection
	spO_2 check	Checking the monitor
C	Pulse check/heart rate	Checking the pulse and the monitor
	Check of skin color	Inspection
	Check of capillary refill time	Inspection and palpation
	Check of blood pressure	(Non-)Invasive blood pressure
	At least one additional big lumen vascular access	Intravenous or intraosseous
D	Pupil check	Inspection
	Re-Evaluation/Anticipation of analgesic medication	Check for pain and planning analgesic therapy
	Re-Evaluation/Anticipation of narcotic medication	Checking state of consciousness and planning narcotic therapy
E	Removing clothes	Cut open or take off
	Temperature check	Auricular or esophageal

**Secondary assessment**	
Full body examination	Inspection and palpation
POCUS examination (eFAST)	Order for the conduct of the examination
Order for full body computer tomography	Order for the conduct of the examination
**Advanced life support**	
CPR Start	Initiation of CPR (as defined in critical action #1) within 10 seconds following the onset of cardiac arrest, or within 10 seconds after completing the mini-thoracotomy if this procedure takes precedence over initiating CPR
Starting the timer	Order or initiation by the team leader
Select team member for time keeping	Order
Chest compressions depth	Order/feedback from the team leader to the team member performing chest compressions. If this feedback is not given, the team member is instructed to deliver inadequate chest compressions
Chest compressions rate	Order/feedback from the team leader to the team member performing chest compressions. If this feedback is not given, the team member is instructed to deliver inadequate chest compressions
Exchange of person performing chest compressions every two minutes	Order/check
Defibrillator placement	Order for placement within 10 seconds of the start of chest compressions and in the correct position
Initial rhythm analysis	Immediate rhythm analysis after placement of the defibrillator
Rhythm analysis at correct times	Rhythm checks according to ERC ALS guidelines
Rhythm analysis duration	No rhythm analysis lasts longer than 5 seconds
Correct recognition of all rhythm analysis	All rhythm analyses must be interpreted correctly (shockable, non-shockable, ROSC)
Pulse checks at correct times	Pulse checks according to ERC ALS guidelines
Continuous ventilation while chest compressions in correct rate (10/min)	Order/feedback from the team leader to the team member at the airway. If this feedback is not given, the team member is instructed to ventilate too quickly (approximately 15-20/min)
FiO_2 set to 1.0	Order/check
Recognition of tension pneumothorax as reversible cause of cardiac arrest	Must be pronounced aloud; or by obvious initiation of mini-thoracotomy after POCUS or clinical observation (ventilatory effort, auscultation, etc.)
Mini-thoracotomy	Order or initiation by the team leader
Medications	Medications, time intervals and dosage according to ERC ALS guidelines

Arterial blood gas	Order or initiation by the team leader (if not already done in the primary/secondary assessment)
Check of possible reversible causes	Check according to ERC guidelines

CPR = cardiopulmonary resuscitation, eFAST = extended focused assessment with sonography for trauma, POCUS = point-of-care ultrasound

Table 2 provides a comprehensive list of items that are deemed protocol deviations until the necessary action is explicitly carried out or verbally communicated in its entirety. For an action or order to be considered timely in this context, it must be completed within the corresponding chronological category outlined in Table 2, which includes Primary Assessment → Secondary Assessment → Advanced Life Support. It is important to note that these determinations are made by an expert in the field, relying on their professional judgment. Each protocol deviation can only be committed once. Actions ordered or performed by the team leader are considered equivalent in this regard.

It should also be noted that all orders and actions can be successfully completed on the first attempt to further standardize the scenario in the setting of this study.

After each scenario, a standardized full-team debriefing will be conducted, focusing on critical actions and protocol deviations. Questionnaire 2 will also need to be filled in by each participant at this point in the study.

3.2.4 Performance assessment

The video recordings, as described in Section 3.2.3, will be analyzed in Phase 4 by blinded experts. The experts will not be involved in the conduct of the study and therefore do not know which group an individual was randomly assigned to in Phase 2. For this reason, the videos presented to the experts are labeled only with an assigned participant ID. To avoid inter-rater variability in the assessments, experts will be given two easy-to-score and two hard-to-score exercise cases to evaluate independently in advance. The results are then discussed among the experts and with the study team to resolve any ambiguities or disagreements and to reach a consensus on further evaluation.

The video recordings themselves will be edited in such a way that the expert is presented with only one compact video of each scenario/assessment, which includes the simultaneous recordings from the cameras pre-installed in the simulation room and the video recording from the participant's perspective through the eye-tracking glasses. These recordings can be viewed and rewound as many times as needed in their final edited form.

In the ALS courses of the ERC, the strategy of mastery learning and mastery testing of skills is used in the context of ALS scenario training and assessment (42), i.e. key objectives are defined that are of particular importance and to which special attention is paid in the assessment. In line with this, a similar focus is also taken in this study. Therefore, the primary outcome measure is the time taken to the most critical action (Table 1; critical action #3), as described in Section 3.2.3. This time (in minutes and

seconds) will be assessed by reviewing the video recordings. The times to the completion of the other critical actions (Table 1; critical actions #1 and #2) will also be measured as secondary outcomes.

If participants do not recognize the underlying condition of the TCA and therefore do not complete all of the critical actions listed in Table 1 within 15 minutes, and therefore only with the assistance of the additional specialist, these times will be considered censored observations (at 15 minutes). The number of such cases will be reported as a secondary outcome.

If patients are declared dead prematurely (before the 15-minute time limit), the time to completion of the respective critical action will also be censored at 15 minutes, and the number of such cases will be reported.

The protocol deviations listed in Table 2 will also be assessed from the video recordings. In this context, untimely is defined as not performed within the following chronological categories:

1. Primary assessment (x – A – B – C – D – E)
2. Secondary assessment
3. ALS according to the ERC's sequence for TCA

The simulated scenario will end at exactly 15 minutes with ROSC achieved if all critical actions have been completed before the allotted time has elapsed. For a positive learning outcome and scenario completion, if the underlying cause is not identified and treated, a simulated specialist in anesthesiology and intensive care will intervene in the scenario at exactly 15 minutes and will provide critical assistance to allow the scenario to still result in ROSC after further 3 minutes. The assessment will end at 15 minutes, regardless of the possible situations described.

All variables collected in Phase 4 will be documented by the experts in the scoring sheet (Additional file #4).

Cognitive load:

Cognitive load will be assessed immediately after the simulation scenarios using the NASA TLX questionnaire, included in Questionnaire 2, the gold standard for measuring subjective workload. The workload score is based on a weighted average of ratings on six sub-dimensions, including mental demand, physical demand, temporal demand, performance, effort, and frustration. All six sub-dimensions (ranging from 1 to 100) are included in the questionnaire. In addition, the global Task Load Index (ranging from 1 to 100), which is defined as the arithmetic mean of all subscales, will be calculated.

Eye-Tracking:

Differences in gaze behavior between the randomized groups in Phase 3 will be assessed in terms of dwell time on different AOIs, number of fixations, and average fixation duration. Defined AOIs are the manikin's head/airway, the manikin's thorax, the vital signs monitor, and the ventilator monitor. The

number of fixations is defined as the number of times participants fixate different AOIs, and the average fixation duration is the average time for fixations within an AOI. The greater the average fixation duration, the greater the level of cognitive engagement.

Furthermore, the relative time when no AOI is illustrated will be analyzed.

3.3 Sample size

A sample size of 30 in each group will have 80% power to detect a probability of at least 0.71 that an observation in Group 1 is less with respect to the primary outcome variable than an observation in Group 2 using a Wilcoxon (Mann-Whitney) rank-sum test with a 0.05 two-sided significance level.

Based on an expected drop-out rate of 10%, 67 participants will be recruited to participate in this study.

3.4 Data collection

Data will be collected from Questionnaires 1 and 2 and scoring sheets. These data will be stored in Microsoft Excel spreadsheets on a secure computer owned by the Department of Anesthesia, Intensive Care Medicine and Pain Medicine at MedUni Vienna, with access restricted to study team members. The physical files will be collected in a folder that will be stored in a locked office in an additional locked box that only study team members will have access to.

All videos will be stored on the same cloud server only in final edited and thus pseudonymized form.

3.4.1 Variables

Table 3. Variables to be collected in this study

Variable	Data source	Notes
**Baseline characteristics**		
Age	Questionnaire 1	<24; 24 – 27; 28 – 29; 30 – 32; >32 (in years)
Gender	Questionnaire 1	Male/female/divers/inter/ open/none
Knowledge of German	Questionnaire 1	First language/fluent/basic
Experience	Questionnaire 1	Junior physician (common-trunk) or first-year resident (anesthesia/intensive care; emergency medicine; traumatology)
Previous participation in ERC ALS courses	Questionnaire 1	Yes/no

Previous participation in certified advanced trauma courses (PHTLS, ETC, ATLS)	Questionnaire 1	Yes/no
Real Life in-hospital CPR experience	Questionnaire 1	5-point Likert scale
Real life out-of-hospital CPR experience	Questionnaire 1	5-point Likert scale
Real life in-hospital trauma management experience	Questionnaire 1	5-point Likert scale
Real life out-of-hospital trauma management experience	Questionnaire 1	5-point Likert scale
Openness to Experience Score (HEXACO subscale)	Questionnaire 1	1 to 5
Technology Readiness Score	Questionnaire 1	12 to 60
Experience with non-VR video games within the last 12 months	Questionnaire 1	5-point Likert scale
Experience with VR video games within the last 12 months	Questionnaire 1	5-point Likert scale
Experience with non-VR video games at the age of 6 to 18 years	Questionnaire 1	5-point Likert scale
Experience with VR video games at the age of 6 to 18 years	Questionnaire 1	5-point Likert scale
Desire for more digital tools in medical education	Questionnaire 1	5-point Likert scale
**Primary outcome**		
Time to critical action #3	Scoring sheet	Minutes : seconds
**Secondary outcomes**		
Recognition and treatment of the underlying condition	Scoring sheet	Yes/no
Patient declared dead prematurely	Scoring sheet	Yes/no
Times to critical actions #1 and #2	Scoring sheet	Minutes : seconds
Protocol deviations	Scoring sheet	Yes/no
NASA-TLX (global and per objective)	Questionnaire 2	0 to 100 points
Eye-Tracking	Scoring sheet	
- Dwell time in AOIs		Minutes : seconds
- Fixation count		Count
- Average fixation duration		Minutes : seconds
- Relative time without AOI		Minutes : seconds
Learning method (participants' impression)		
- Learning progress		

- Level of frustration	Questionnaire 2	5-point Likert scale
- Level of enjoyment		
Participants' confidence in management of TCA	Questionnaires 2	5-point Likert scale
Participants' performance		
- Participants' subjective impression	Questionnaires 2	5-point Likert scale
- Experts' impression	Scoring sheet	5-point Likert scale
VR-related adverse events	Questionnaire 2	Incidence rate
SUS	Questionnaire 2	
- SUS score		1 to 100
- Adjective rating scale		7-point Likert scale
**Drop-out variables**		
Training/studying with other non-study learning resources	Questionnaire 2	Yes/no
Insufficient preparation with assigned learning method		Yes/no

ALS = advanced life support; AOI = area of interest; ATLS = advanced trauma life support; CPR = cardiopulmonary resuscitation; ERC = European Resuscitation Council; ETC = European Trauma Course; NASA-TLX = National Aeronautics and Space Administration Task Load Index; PHTLS = prehospital trauma life support; SUS = system usability scale; TCA = traumatic cardiac arrest; VR = virtual reality

3.4.2 Data protection

All data collected on study participants will be pseudonymized with a sequential numerical code. Direct references to the identity of these individuals will only be found on the informed consent forms (see Section 4.1) and an Excel sheet with contact information accessible only by study team management.

All videos will be deleted three months after the completion of the study.

Only the study team has access to the data and videos collected. The data is pseudonymized, password-protected, and firewalled. In addition, the data is stored separately from the individual informed consent forms and the Excel spreadsheet containing all contact information for participants. The informed consent forms and the latter Excel spreadsheet are kept secure and only the study management has access to them. Access to the data that directly identifies participants is therefore only available to the study management. In addition, authorized representatives of the study management, who are bound to secrecy, as well as representatives of national and/or international health authorities and ethics committees may have access to these data, as far as this is necessary or required to verify the proper conduct of the study.

All persons who have access to this data are subject to the applicable national data protection regulations and/or the European Union (EU) General Data Protection Regulation (GDPR) when handling the data. The code that allows the pseudonymized data to be associated with you is kept only at the study center.

The data will only be passed on in the pseudonymized or anonymized form. The data will only be used in fully anonymized form for any publications.

According to the GDPR, participants generally have the right of access, rectification, deletion, restriction of processing, data portability and objection, if this does not make the study's objectives impossible or seriously impair them and provided that this does not conflict with other legal provisions.

3.4.3 Data analysis

After data collection and compilation are completed in Microsoft Excel, they will be processed for further statistical analysis. Furthermore, two independent study team members will check the data for plausibility.

3.5 Statistical methods

Continuous data will be reported using median and interquartile range (IQR) if skewed or mean ± standard deviation (SD) if normally distributed. Counts and percentages with 95% confidence intervals (CI) will be given for categorical data.

Primary objective/outcome:

A two-sided generalized Wilcoxon test with a 5% significance level will be used to compare the primary outcome between the randomized groups. Participants who do not identify and treat the underlying condition of TCA will be censored at 15 minutes. Furthermore, if patients are declared dead prematurely, the observation will also be censored at 15 minutes.

Secondary objectives/outcomes:

A two-sided generalized Wilcoxon test will also be used to compare times to critical actions, excluding the primary outcome. The number of participants who did not recognize and treat the underlying condition of the TCA, and the number of participants declaring the patient dead prematurely, will be compared between groups calculating the chi-square test. Protocol deviations will be analyzed separately for each protocol deviation listed in Table 2. The respective frequencies of each protocol deviation will also be compared between groups calculating the chi-square test. Fisher's exact test will be used if one or more expected cell frequencies are less than 5.

In addition, the Wilcoxon rank sum test will also be used to compare the gaze behavior (dwell-time in AOIs, fixation count and average fixation duration, and relative time when no AOI is illustrated) and the NASA-TLX scores (global and per objective) between randomized groups.

Furthermore, a multivariable Cox regression model (including an interaction term) will be used to determine the influence of gender on the primary outcome, and the potential modifying effect of gender on the training effect (= group effect). The HEXACO subscale and the Technology Readiness Score will also be included in the multivariable model to adjust for potential imbalances between the randomized groups.

The following Spearman correlation coefficients will be calculated corresponding to the remaining secondary objectives:

- Correlation of 5-point Likert scales between the frequency of playing classic video games and the primary outcome

5-point Likert scales, the incidence rate of VR-related AEs, and the SUS scores will be reported using descriptive statistics only.

Observations with missing data in the primary outcome measure will be excluded from statistical analyses. Boxplots will be used to illustrate calculations. A two-sided p-value of < 0,05 will be considered statistically significant.

Due to the singular primary objective, no correction for multiple testing will be made. All secondary objectives are exploratory in nature and will be used to generate further hypotheses.

3.6 Materials

3.6.1 VR Headset

For this study, participants in the intervention group will be provided with a VR headset, the HTC VIVE Focus 3 with two controllers. The appropriate software will be pre-installed and ready to use.

A study team member will be present during Phase 2 to assist with any technical difficulties.

3.6.2 Eye-tracking glasses

Video recordings will also be made from the perspective of the team leader using eye-tracking glasses, Tobi Pro Glasses 3.0, in addition to the conventional cameras in the simulation center.

Furthermore, eye movements will be recorded at a rate of 50 Hz. The data will be analyzed using the Tobii Pro Glasses Analyzer software. The area of visual attention detected by the glasses is marked with a circle in the video recordings.

3.6.3 E-learning platform

Moodle, the e-learning platform of the MedUni Vienna, is used to teach relevant content in the control group and is available to participants free of charge.

3.6.4 Video editing

Video editing will be done using the DaVinci Resolve 18 software.

4 PROTECTION OF HUMAN SUBJECTS

4.1 Informed consent

Study participants will be recruited as described in Section 3.1. Their participation is therefore voluntary. Prior to participation in the study, a member of the study team will provide detailed information about the potential benefits and disadvantages, procedure, and data used in this study. This explanation will also be recorded in writing on the informed consent form (Additional file #1). Study participation will only occur after written informed consent has been given.

4.2 Patient withdrawal

Participants may withdraw from the study at any time without giving a reason.

4.3 Ethical considerations and risk-benefit-evaluation

Since this is a comparative study of different learning methods, there is no risk to the study participants. The only risk is the disclosure of sensitive information about them, which is minimized by pseudonymization and access restrictions.

Further hypotheses can be generated from the results of this study.

REFERENCES

1. Vianen NJ, Van Lieshout EMM, Maissan IM, Bramer WM, Hartog DD, Verhofstad MHJ, et al. Prehospital traumatic cardiac arrest: a systematic review and meta-analysis. Eur J Trauma Emerg Surg. 2022 Aug;48(4):3357–72.
2. Chan PS, Nichol G, Krumholz HM, Spertus JA, Nallamothu BK, American Heart Association National Registry of Cardiopulmonary Resuscitation (NRCPR) Investigators. Hospital variation in time to defibrillation after in-hospital cardiac arrest. Arch Intern Med. 2009 Jul 27;169(14):1265–73.
3. Chan PS, Krumholz HM, Nichol G, Nallamothu BK, American Heart Association National Registry of Cardiopulmonary Resuscitation Investigators. Delayed time to defibrillation after in-hospital cardiac arrest. N Engl J Med. 2008 Jan 3;358(1):9–17.
4. Abella BS, Alvarado JP, Myklebust H, Edelson DP, Barry A, O'Hearn N, et al. Quality of cardiopulmonary resuscitation during in-hospital cardiac arrest. JAMA. 2005 Jan 19;293(3):305–10.
5. Abella BS, Sandbo N, Vassilatos P, Alvarado JP, O'Hearn N, Wigder HN, et al. Chest compression rates during cardiopulmonary resuscitation are suboptimal: a prospective study during in-hospital cardiac arrest. Circulation. 2005 Feb 1;111(4):428–34.
6. Søreide E, Morrison L, Hillman K, Monsieurs K, Sunde K, Zideman D, et al. The formula for survival in resuscitation. Resuscitation. 2013 Nov;84(11):1487–93.
7. Morrison LJ, Neumar RW, Zimmerman JL, Link MS, Newby LK, McMullan PW, et al. Strategies for improving survival after in-hospital cardiac arrest in the United States: 2013 consensus recommendations: a consensus statement from the American Heart Association. Circulation. 2013 Apr 9;127(14):1538–63.
8. Okuda Y, Bryson EO, DeMaria S, Jacobson L, Quinones J, Shen B, et al. The utility of simulation in medical education: what is the evidence? Mt Sinai J Med. 2009 Aug;76(4):330–43.
9. Lopreiato JO, Sawyer T. Simulation-based medical education in pediatrics. Acad Pediatr. 2015 Apr;15(2):134–42.
10. Jeffers J, Eppich W, Trainor J, Mobley B, Adler M. Development and Evaluation of a Learning Intervention Targeting First-Year Resident Defibrillation Skills. Pediatr Emerg Care. 2016 Apr;32(4):210–6.
11. McGrath JL, Taekman JM, Dev P, Danforth DR, Mohan D, Kman N, et al. Using virtual reality simulation environments to assess competence for emergency medicine learners. Acad Emerg Med. 2018 Feb;25(2):186–95.
12. Louie MC, Chang TP, Grundmeier RW. Recent advances in technology and its applications to pediatric emergency care. Pediatr Clin North Am. 2018 Dec;65(6):1229–46.
13. Abu-Zidan FM. Advanced trauma life support training: How useful it is? World J Crit Care Med. 2016 Feb 4;5(1):12–6.

14. Mohammad A, Branicki F, Abu-Zidan FM. Educational and clinical impact of Advanced Trauma Life Support (ATLS) courses: a systematic review. World J Surg. 2014 Feb;38(2):322–9.

15. Greif R, Lockey A, Breckwoldt J, Carmona F, Conaghan P, Kuzovlev A, et al. European Resuscitation Council Guidelines 2021: Education for resuscitation. Resuscitation. 2021 Apr;161:388–407.

16. Lerner D, Mohr S, Schild J, Göring M, Luiz T. An Immersive Multi-User Virtual Reality for Emergency Simulation Training: Usability Study. JMIR Serious Games. 2020 Jul 31;8(3):e18822.

17. Chang TP, Weiner D. Screen-Based Simulation and Virtual Reality for Pediatric Emergency Medicine. Clin Pediatr Emerg Med. 2016 Sep;17(3):224–30.

18. Nas J, Thannhauser J, Vart P, van Geuns R-J, Muijsers HEC, Mol J-Q, et al. Effect of Face-to-Face vs Virtual Reality Training on Cardiopulmonary Resuscitation Quality: A Randomized Clinical Trial. JAMA Cardiol. 2020 Mar 1;5(3):328–35.

19. Lauridsen KG, Løfgren B, Brogaard L, Paltved C, Hvidman L, Krogh K. Cardiopulmonary resuscitation training for healthcare professionals: A scoping review. Simul Healthc. 2021 Oct 13;

20. Greif R, Bhanji F, Bigham BL, Bray J, Breckwoldt J, Cheng A, et al. Education, implementation, and teams: 2020 international consensus on cardiopulmonary resuscitation and emergency cardiovascular care science with treatment recommendations. Resuscitation. 2020 Nov;156:A188–239.

21. Josey K, Smith ML, Kayani AS, Young G, Kasperski MD, Farrer P, et al. Hospitals with more-active participation in conducting standardized in-situ mock codes have improved survival after in-hospital cardiopulmonary arrest. Resuscitation. 2018 Dec;133:47–52.

22. Perkins GD, Fullerton JN, Davis-Gomez N, Davies RP, Baldock C, Stevens H, et al. The effect of pre-course e-learning prior to advanced life support training: a randomised controlled trial. Resuscitation. 2010 Jul;81(7):877–81.

23. Thorne CJ, Lockey AS, Bullock I, Hampshire S, Begum-Ali S, Perkins GD, et al. E-learning in advanced life support--an evaluation by the Resuscitation Council (UK). Resuscitation. 2015 May;90:79–84.

24. Au K, Lam D, Garg N, Chau A, Dzwonek A, Walker B, et al. Improving skills retention after advanced structured resuscitation training: A systematic review of randomized controlled trials. Resuscitation. 2019 May;138:284–96.

25. Lau Y, Nyoe RSS, Wong SN, Ab Hamid ZB, Leong BS-H, Lau ST. Effectiveness of digital resuscitation training in improving knowledge and skills: A systematic review and meta-analysis of randomised controlled trials. Resuscitation. 2018 Oct;131:14–23.

26. Lockey AS, Dyal L, Kimani PK, Lam J, Bullock I, Buck D, et al. Electronic learning in advanced resuscitation training: The perspective of the candidate. Resuscitation. 2015 Dec;97:48–54.

27. Arithra Abdullah A, Nor J, Baladas J, Tg Hamzah TMA, Tuan Kamauzaman TH, Md Noh AY, et al. E-learning in advanced cardiac life support: Outcome and attitude among healthcare professionals. Hong Kong Journal of Emergency Medicine. 2020 Nov;27(6):328–33.

28. Tramèr L, Becker C, Schumacher C, Beck K, Tschan F, Semmer NK, et al. Association of self-esteem, personality, stress and gender with performance of a resuscitation team: A simulation-based study. PLoS ONE. 2020 May 14;15(5):e0233155.

29. Amacher SA, Schumacher C, Legeret C, Tschan F, Semmer NK, Marsch S, et al. Influence of gender on the performance of cardiopulmonary rescue teams: A randomized, prospective simulator study. Crit Care Med. 2017 Jul;45(7):1184–91.

30. Soar J, Böttiger BW, Carli P, Couper K, Deakin CD, Djärv T, et al. European Resuscitation Council Guidelines 2021: Adult advanced life support. Resuscitation. 2021 Apr;161:115–51.

31. Lott C, Truhlář A, Alfonzo A, Barelli A, González-Salvado V, Hinkelbein J, et al. European Resuscitation Council Guidelines 2021: Cardiac arrest in special circumstances. Resuscitation. 2021 Apr;161:152–219.

32. Deutsche Gesellschaft für Unfallchirurgie e.V. S3 - Leitlinie Polytrauma/Schwerverletzten - Behandlung (AWMF Registernummer 187 - 023). AWMF [Internet]. 2022 Dec 31 [cited 2023 Apr 15];Version 4.0. Available from: https://www.awmf.org/leitlinien/detail/ll/187-023.html

33. Lee K, Ashton MC. Psychometric Properties of the HEXACO-100. Assessment. 2018 Jul;25(5):543–56.

34. HEXACO-PI-R Materials for Researchers [Internet]. [cited 2023 Jun 27]. Available from: http://hexaco.org/hexaco-inventory

35. Neyer FJ, Felber J, Gebhardt C. Entwicklung und Validierung einer Kurzskala zur Erfassung von Technikbereitschaft. Diagnostica. 2012 Apr;58(2):87–99.

36. Bangor A, Kortum P, Miller J. Determining what individual SUS scores mean: Adding an adjective rating scale. Journal of usability studies. 2009;

37. Abulfaraj MM, Jeffers JM, Tackett S, Chang T. Virtual Reality vs. High-Fidelity Mannequin-Based Simulation: A Pilot Randomized Trial Evaluating Learner Performance. Cureus. 2021 Aug 11;13(8):e17091.

38. Medical University of Vienna, ITSC. The Randomizer [Internet]. [cited 2023 Mar 23]. Available from: https://cemsiis.meduniwien.ac.at/en/kb/science-research/software/randomizer/

39. Lauridsen KG, Watanabe I, Løfgren B, Cheng A, Duval-Arnould J, Hunt EA, et al. Standardising communication to improve in-hospital cardiopulmonary resuscitation. Resuscitation. 2020 Feb 1;147:73–80.

40. Virtuelle Lern- und Arbeitswelten – in 3D, ohne Limits | TriCAT [Internet]. [cited 2023 Apr 1]. Available from: https://tricat.net/

41. Stanney KM, Kennedy RS, Drexler JM. Cybersickness is not simulator sickness. Proceedings of the Human Factors and Ergonomics Society Annual Meeting. 1997 Oct;41(2):1138–42.

42. Ringsted C, Lippert F, Hesselfeldt R, Rasmussen MB, Mogensen SS, Frost T, et al. Assessment of Advanced Life Support competence when combining different test methods--reliability and validity. Resuscitation. 2007 Oct;75(1):153–60.

APPENDIX

Document reference number	Title
Additional file #1	Informed consent
Additional file #2	Questionnaire 1
Additional file #3	Questionnaire 2
Additional file #4	Scoring
Additional file #5	Bulletin board posting
